# Agreement between mothers’, fathers’, and teachers’ ratings of behavioural and emotional problems in 3–5-year-old children

**DOI:** 10.1371/journal.pone.0206752

**Published:** 2018-11-01

**Authors:** Elisabet Fält, Thomas Wallby, Anna Sarkadi, Raziye Salari, Helena Fabian

**Affiliations:** 1 Child Health and Parenting (CHAP), Department of Public Health and Caring Sciences, Uppsala University, Uppsala, Sweden; 2 Research Enhancing Adolescent and Child Health (REACH), Department of Women’s and Children’s Health, Uppsala University, Uppsala, Sweden; TNO, NETHERLANDS

## Abstract

**Background:**

The Strengths and Difficulties Questionnaire (SDQ), a valid and reliable instrument for measuring children’s mental health, is available in parent- and teacher versions, making it an ideal tool for assessing behavioural and emotional problems in young children. However, few studies have evaluated inter-parent agreement on the SDQ, and in most studies on SDQ agreement, parent scores are either provided by only one parent or have been combined into one parent score. Furthermore, studies on SDQ inter-rater agreement usually only reflect degree of correlation, leaving the agreement between measurements unknown. The aim of the present study was therefore to examine both degree of correlation and agreement between parent and teacher SDQ reports, in a community sample of preschool-aged children in Sweden.

**Methods:**

Data were obtained from the Children and Parents in Focus trial. The sample comprised 4,469 children 3–5-years-old. Mothers, fathers and preschool teachers completed the SDQ as part of the routine health check-ups at Child Health Centres. Inter-rater agreement was measured using Pearson correlation coefficient and intraclass correlation (ICC).

**Results:**

Results revealed poor/fair agreement between parent and teacher ratings (ICC 0.25–0.54) and good/excellent agreement between mother and father ratings (ICC 0.66–0.76). The highest level of agreement between parents and teachers was found for the hyperactivity and peer problem subscales, whereas the strongest agreement between parents was found for the hyperactivity and conduct subscales.

**Conclusions:**

Low inter-rater agreement between parent and teacher ratings suggests that information from both teachers and parents is important when using the SDQ as a method to identify mental health problems in preschool children. Although mothers and fathers each provide unique information about their child’s behaviour, good inter-parent agreement indicates that a single parent informant may be sufficient and simplify data collection.

## Introduction

Early identification and treatment of mental health problems in young children can have immediate effects on the child´s quality of life and benefit the child’s health in a long-term perspective, as emotional and behavioural problems left undetected tend to become persistent or increase in severity [1, 2]. Identifying children with mental health problems, and addressing these problems early on will also result in socio-economic benefits [3, 4]. Signs of behavioural and emotional problems may be highly situational; thus, a multi-informant approach is considered as the best practice for assessment of behavioural and emotional problems in children [5].

In Sweden, Child Health Services (CHS) offer health and developmental checkups at Child Health Centres (CHCs) by public health nurses and general practitioners to all parents with children aged six and under. The routine health checkups are free of charge and occur frequently during the child’s first 18 months and become annual visits once the child turns three. Although one of the objectives of the CHS is to detect developmental and mental health problems in children [6], evidence-based methods are not used for that purpose at the routine health checkups for 3–5-year-olds. Instead, the clinical assessment relies on parent’s description of their children’s everyday functioning, and preschool teachers are consulted only if parents express concerns regarding their children. This is so despite that (a) teachers are recognised as an important informant in identifying children with mental health problems [7], (b) in Sweden, more than nine out of ten 3–5-year-old children attend preschool [8], and (c) the Swedish preschool is characterised by high quality and well-educated staff.

As part of a population-based cluster-randomised trial [9] in Uppsala, Sweden, a method of information sharing between CHS, preschool and parents was introduced. The information sharing method was performed using the Strengths and Difficulties Questionnaire (SDQ) [10]. SDQ is a well-known instrument for measuring children’s mental health, available in both parent- and teacher versions. The SDQ is brief, commonly used internationally and considered to be an instrument with good psychometric properties [10–12]. However, psychometric properties are not fixed values but rather measures of the instrument when applied to certain populations for a specific purpose [13, 14]. Previous research has established cross-cultural differences in the reliability of the SDQ [15, 16]. Hence, in order to use the SDQ as a method to identify mental health problems in children at CHCs, the psychometric properties of the SDQ in the specific cultural context and population need to be established. In addition, given that parent and teacher ratings are often inconsistent [5], it is crucial to provide clear guidelines regarding how clinicians should deal with conflicting information. In order to develop adequate guidelines, it is necessary to test the relation between the ratings done by different informants in the specific population.

Relations between variables are often studied using two concepts: correlation and agreement. Although related, these concepts reflect different types of association and, thus, require the use of different statistical techniques. Pearson’s r measures linear correlation i.e. consistency, between different raters. However, Pearson correlations do not provide information about the extent to which the raters’ individual scores actually match. This is because two variables can be highly correlated even when they differ greatly as long as one variable is consistently higher or lower than the other. Agreement analysis, on the other hand, requires both correlation and coincidence of scores. The Intraclass Correlation Coefficient (ICC) is a statistical test of absolute agreement (or consistency) between continuous variables [17]. High ICC values indicate that the two variables have very similar values.

A meta-analysis [5] by Achenbach et al. (1987) reported fairly low (Pearson product moment) correlations between parent and teacher ratings of children’s behavioural and emotional problems (0.28) but higher correlations between parents’ ratings (0.60). The meta-analysis calculated the mean inter-rater correlation for 119 studies reporting inter-rater agreement of children’s (aged 1.5–19) behavioural and emotional problems. The mean correlations between different types of informants (e.g. parents and teachers) reported by Achenbach et al. (1987) have been re-established in a later meta-analysis by De Los Reyes et al. (2015) [18]. This more recent meta-analysis included 341 studies published between 1989 and 2014, reporting estimates of correlation between the reports of different informants on children’s (at or under 18 years) mental health. Hence, it is known that correlations between parent and teacher reports on children’s behavioural and emotional problems are modest, and findings from community-based studies have indicated that parents and teachers often disagree in their assessments of children’s psychosocial problems [19–21]. However, the inter-rater correlations for the SDQ between parents and teachers are higher than average values reported for similar questionnaires [10, 11, 22]. A review [11] reported inter-rater correlations for the SDQ between 0.26 and 0.47, where all the subscale correlations except the prosocial scale were higher than the meta-analytic mean reported by Achenbach et al. [5] (r = 0.27), and the best correlation was found for the hyperactivity scale.

Multi-informant approach is emphasised when using SDQ to identify mental health problems among children [11, 23, 24]. However, few studies have evaluated inter-parent agreement on the SDQ [25]; moreover, in most studies on SDQ inter-rater agreement, parent scores are either provided by only one parent or have been combined into one parent score in the analyses. This is surprising as previous research on inter-parent agreement suggests that there are differences between mother and father reports on behavioural and emotional problems [25–27]. Furthermore, studies on SDQ inter-rater agreement usually only reflect degree of correlation (Pearson/Spearman correlation coefficients), leaving the agreement between measurements unknown. As mentioned before, intraclass correlation coefficient (ICC) is an index reflecting both degree of correlation and agreement between measurements [28, 29]. To the best of our knowledge, ICC has only been used in three studies to measure agreement of the SDQ [30–32]. However, in these studies, ICC was calculated only between parent–teacher ratings, i.e. not specifically teacher–father or teacher–mother ratings and also not between parents; in addition, two of the studies were based on the same sample.

Although there is no gold-standard in handling inter-rater discrepancies, evaluation of inter-rater agreement is important in all contexts in which multi-informant approach is used and decisions on how clinicians are to interpret differences in reports of the same child’s functioning need to be made. Providing clinicians with research-based knowledge regarding e.g. expected/unexpected levels of informant (dis)agreement for each SDQ subscale could help them to judge the importance of the discrepancies they observe. It is also important to examine agreement between parent and teacher reports regarding their perceived impact of the child’s behavioural and emotional problems since this is of crucial importance to clinical decision-making. However, relatively few studies have compared the assessed impact of the problems alongside ratings of psychiatric symptoms in the problem subscales [33].

The aim of the present study was therefore to test the inter-rater agreement between parents, and parent and teacher reports, respectively, including impact, using SDQ in 3–5-year-old children visiting CHCs.

## Methods

### Data collection

The present study was part of the Children and Parents in Focus trial, a study focusing on preventing behavioural problems in children [9]. All parents of 3, 4 and 5-year-old children born between 2008–2011, enrolled at CHCs within Uppsala County, were invited to participate. As part of the trial, parent- and teacher reports of the SDQ were implemented as part of the routine checkup at 3, 4 and 5 years of age. All of the CHCs within Uppsala County were invited to participate in the study the first year: in total, 43 out of 45 CHCs participated. Of 22 CHCs in Uppsala Municipality (invited the second year), 20 participated.

CHC-nurses attached study information, consent form and three sets of SDQ (one for each of the child’s legal custodians and one for the teacher) to the invitation letter that parents routinely receive about three weeks prior to their 3–5-year-old children’s routine checkup. In the written study information, parents were asked to give the questionnaire to the child’s preschool teachers, and teachers were asked to complete the questionnaire and then post it back to the child’s CHC-nurse in a prepaid envelope. Parents were asked to complete their questionnaires and return them together with their consent form when attending the child’s visit at the CHC. During the checkup, the nurse reviewed the questionnaires.

Translated versions of the study information and the questionnaires were provided in three languages commonly spoken by migrant populations in Sweden: Arabic, Somali and English. Parents who were unable to complete the SDQ in Swedish or any of the mentioned languages were excluded from the trial. The number of informants varied from one to three, since parents were free to decide whether both parents and/or the preschool were to complete the SDQ.

#### Sample

The study sample for the present study included 4,469 children 3–5-years old (51.4% boys), born between 2008–2011 who participated in the Children and Parents in Focus trial, and had been assessed by two, and only two (n = 1,509) or all three (n = 2,960) informants. For those children who were present at both the first and second year of the trial, only the first assessment was included in the analyses for the present study, as the second assessment was not an independent observation. The study sample for the present study made up 24.9% of the total number of children in the *county* during the first year of the trial (n = 10,160) and 30.3% of the total number of children in the *municipality* during the second year of the trial (n = 6,419). In total, there were 4,329 SDQ reports from mothers, 3,855 from fathers and 3,714 reports from teachers. Socio-demographic data for the participating children and parents are provided in Table 1. Children with an SDQ report from only one informant (n = 1,167) were excluded for the purposes of this paper.

**Table 1:** Socio-demographic characteristics of the participating children and parents (n = 4,469).

	Frequency	Percent
**Child’s gender**		
Female	2,170	48.6
Male	2,299	51.4
**Child’s age (years)**		
3	1,331	29.8
4	1,366	30.6
5	1,772	39.7
**Living arrangement**		
Original family (child lives with both parents)	3,916	87.6
Joint physical custody	234	5.2
Child lives with one parent	74	1.7
Other/No information	240	5.5
**Mother's country of birth**		
Sweden	3,674	87.3
Other	533	12.7
**Father's country of birth**		
Sweden	3,304	88.4
Other	435	11.6
**Mother's relationship status**		
Married/cohabiting	3,973	93.9
Single	260	6.1
**Father's relationship status**		
Married/cohabiting	3,614	96.1
Single	148	3.9
**Mother's education**		
Secondary school	96	2.3
High school	1,089	26.2
University	2,975	71.5
**Father's education**		
Secondary school	119	3.2
High school	1,326	35.9
University	2,247	60.9

#### Ethics

The trial was approved by the Regional Ethical Review Board in Uppsala (Dnr 2012/437). The parents were provided with written study information sheets together with the questionnaires, and the parents or legal guardians of all participating children provided written informed consent on behalf of their children.

#### The strengths and difficulties questionnaire

The SDQ is a valid instrument for identification of mental health problems in community-based samples [34–37]. The questionnaire takes about five to ten minutes to complete and is available for 3–16-year-olds. The validity of the Swedish version of the SDQ (SDQ-Swe) has been assessed in 5–15-year-old children [12], and norms for parent reports are available for 6–10-year-olds [38]. Data (means, standard deviations and 90th/10th percentile) from a norming study on Swedish 2–5-year-olds have been presented [39], but not yet published.

The SDQ consists of 25 items classified into five subscales, consisting of four problem subscales (emotional symptoms, conduct problems, hyperactivity/inattention and peer problems) and one subscale on prosocial behaviour [10]. Each subscale consists of five items scored on a 3-point Likert scale with 0 = not true, 1 = somewhat true and 2 = certainly true. Subscale scores range between 0 and 10, while the total difficulties score from the four problem subscales (total difficulties score) ranges between 0 and 40.

SDQ is also available in versions with an impact supplement [40], which comprises eight items capturing perceived difficulties: chronicity, overall distress, social impairment and burden. The impact supplement provides information central to clinical decision-making in current diagnostic classification systems [10, 41]. In the present study, the impact supplement’s first item was used as a supplementary measure. The first item is the only impact item included in the teacher SDQs administered in the present study. Hence, for evaluating inter-rater agreement, impact scores could only be generated from this specific question. The item asks whether the informant (parent or teacher) thinks that the child has difficulties in one or more of the subscale areas, and is scored on a 4-point Likert scale with 0 = no; 1 = Yes, minor difficulties; 2 = Yes, definite difficulties; and 3 = Yes, severe difficulties. According to Goodman [40], it makes no sense to ask informants about chronicity, distress, impairment or burden when they perceive no difficulties. In those cases, informants are told to leave out the other items, which are subsequently coded as zero. This supports the use of only the first impact question when SDQ is used in a community sample where most children are healthy and a brief measure is often prioritised. This is also why the impact score was dichotomised by classifying children rated with 0 = not case and 1, 2, 3 = case.

The preschool representatives did not approve the wording in all SDQ items. Thus, in order to use SDQ-Swe in the context of preschools, minor modifications in the wording of three items had to be made (Table 2). Research has shown that even seemingly minor changes to structured instruments may have large effect on mean scores [42]. However, the need to modify interventions to fit the host setting is a critical step in implementation [43] and without the item modifications using the SDQ in the preschool context would have been impossible. Construct validity was assessed using confirmatory factor analysis, and results showed good fit for all informants [44]. This indicates that the factor structure of the modified SDQ is comparable to the original version.

**Table 2:** Modification of items in SDQ-Swe.

Item	Standard SDQ item	Modified SDQ item
**7**	Generally obedient, usually does what adults request	Usually does what adults request
	Som regel lydig, följer vanligtvis vuxnas uppmaningar (In Swedish)	Följer vanligtvis vuxnas uppmaningar (In Swedish)
**17**	Kind to younger children	Considerate of younger children
	Snäll mot yngre barn (In Swedish)	Omtänksam mot yngre barn (In Swedish)
**22**	Can be spiteful to others	Can behave spitefully to others
	Kan vara elak mot andra (In Swedish)	Kan bete sig illa mot andra (In Swedish)

#### Outcome variables

The main outcome variables in the present study were the SDQ score at subscale level, the total difficulties score (continuous variables) plus a dichotomisation of the impact item (coded either 0 or 1). Missing data were handled in concordance with the guidelines recommended by the SDQ developers (sdqinfo.com). Accordingly, if only one or two items were missing in a subscale, the subscale score was generated by scaling up pro-rata, and if three or more items in a subscale were missing then that subscale was excluded.

### Statistical analyses

Means and standard deviations were calculated to summarise the SDQ scores reported by parents and teachers. One-way repeated measures ANOVAs were performed to analyse differences between parent and teacher mean scores for each of the five subscales and for the total difficulties. In these analyses, we also examined the magnitude of the effect sizes using Partial Eta Squared (η^2^_*p*_). The effect sizes were interpreted using the cut-offs presented by Cohen in 1988 [45] where η^2^_*p*_ = .01, .06, and .14 represent small, medium and large effect sizes respectively.

To evaluate the inter-rater agreement, two different approaches were used:

1. Pearson correlations—to enable comparisons with the meta-analytic mean [5] reported by Achenbach et al. (1987), as well as other published studies.
2. Intraclass correlation coefficients (ICC)—to complement the Pearson correlation analyses by reflecting both degree of correlation and agreement between measurements.

Inter-rater agreement (between mothers and fathers, mothers and teachers, fathers and teachers) based on continuous scores, was examined by Pearson’s correlation coefficient. The level of agreement of the SDQ ratings was evaluated in all subscales and on the total difficulties score. Pearson’s parametric test was used, although there were deviations from the normal distribution in the data for all subscales (Shapiro-Wilk test, *p* < 0.01; W = 0.549–0.945), as the meta-analytic mean reported by Achenbach et al. [5] was based on Pearson’s correlations. The pattern of the findings was the same when Spearman nonparametric correlations were used. Hence, we used Pearson’s correlations throughout the study to allow for comparability with the Achenbach study.

Intraclass correlation coefficients (ICCs) were determined to study the inter-rater agreement at subscale and total scale level (continuous) and on the SDQ impact score (dichotomised scores). The two-way random average measures form for ICC was used, as it is recommended for evaluating rater-based clinical assessment methods [28]. Absolute agreement was selected when calculating the ICC. The level of agreement is preferable evaluated by the ICC estimate’s 95% confidence interval [28]. According to often cited guidelines [46], the following classification of ICC values were used: ICC values < 0.40 = poor agreement, values between 0.40 and 0.59 = fair agreement, values between 0.60 and 0.74 = good agreement, and values > 0.75 = excellent agreement.

The Fisher’s z’ transformation [47] was used to test the significance of the difference between the correlation coefficients. Correlations computed between different informants were compared to each other as well as to the values reported by Achenbach et al. (1987). The p-value was set at 0.001, considering the large sample size. Data were analysed using SPSS version 22 [48].

## Results

### Descriptive overview

The parents in the attained sample were more likely to have been born in Sweden, cohabiting and also had higher education level compared to the target population in Uppsala Municipality (*p* < 0.001 for all). The mean SDQ scores for parent and teacher SDQ scores are presented in Table 3. One-way repeated measures ANOVAs showed that mothers generally reported significantly lower levels of problems compared to fathers, and teachers generally reported significantly lower levels of problems compared to both mothers and fathers. However, while effect sizes were mostly small for differences between mothers and fathers, for the differences between teachers and parents, effect sizes were large for total difficulties as well as two of the subscales (emotional symptoms and conduct problems).

**Table 3:** Mean scores, standard deviations and effect sizes for mother, father and teacher ratings of 3-, 4-, and 5–year old children.

	Mother and father ratings (n = 3,712)				Mother and teacher ratings (n = 3,574)				Father and teacher ratings (n = 3,100)			
	Mother	Father	Mean difference		Mother	Teacher	Mean difference		Father	Teacher	Mean difference	
	Mean (SD)	Mean (SD)	*P*	η^2^_*p*_	Mean (SD)	Mean (SD)	*P*	η^2^_*p*_	Mean (SD)	Mean (SD)	*P*	η^2^_*p*_
**Emotional symptoms**	1.09 (1.31)	1.13 (1.28)	.056	.001	1.12 (1.35)	0.52 (1.02)	.000	.136	1.13 (1.29)	0.50 (1.00)	.000	.153
**Conduct problems**	1.89 (1.68)	2.07 (1.71)	.000	.012	1.92 (1.70)	0.98 (1.55)	.000	.195	2.06 (1.71)	0.97 (1.52)	.000	.230
**Hyperactivity**	2.01 (2.00)	2.24 (1.99)	.000	.016	2.04 (2.01)	1.68 (2.20)	.000	.023	2.25 (1.98)	1.63 (2.17)	.000	.059
**Peer problems**	0.70 (1.14)	0.84 (1.18)	.000	.016	0.70 (1.16)	0.52 (1.09)	.000	.019	0.83 (1.19)	0.50 (1.05)	.000	.058
**Prosocial behaviour**	8.29 (1.71)	8.07 (1.73)	.000	.017	8.27 (1.72)	8.16 (2.12)	.005	.002	8.07 (1.72)	8.20 (2.10)	.004	.003
**Total difficulties**	5.68 (4.16)	6.27 (4.24)	.000	.026	5.77 (4.27)	3.70 (4.20)	.000	.160	6.27 (4.27)	3.60 (4.13)	.000	.219

SDQ = Strengths and Difficulties Questionnaire. P values show the Wilks’ Lambda, effect sizes show the Partial Eta Squared (η^2^_*p*_). Magnitude of η^2^_*p*_: .01 = small, .06 = medium, and .14 = large

### Inter-rater agreement

Inter-rater correlations (Pearson’s correlation coefficient) between mother, father and teacher SDQ reports, at subscale level and total difficulties score, are presented in Table 4. Values for the inter-rater agreement (Intraclass correlation coefficients, ICC) between mother, father and teacher SDQ reports, at subscale level and total difficulties score and on the dichotomised impact item are presented in Table 5. The ICCs between parent and teacher ratings are also presented by age in Table 6, and by gender in Table 7. The confidence intervals indicate that ICCs are generally similar across child age and gender. Correlations (Pearson and ICC) were statistically significant (*p* < 0.001) for all scales.

**Table 4:** Pearson inter-rater correlations for SDQ scores.

SDQ scales	Mother and father (n = 3,712)	Mother and teacher (n = 3,574)	Father and teacher (n = 3,100)
**Emotional symptoms**	.53 (.52)	.22 (.21)	.18 (.16)
**Conduct problems**	.57 (.56)	.32 (.28)	.25 (.23)
**Hyperactivity**	.61 (.55)	.40 (.34)	.32 (.27)
**Peer problems**	.51 (.46)	.32 (.25)	.30 (.23)
**Prosocial behaviour**	.53 (.52)	.27 (.25)	.21 (.19)
**Total difficulties**	.62 (.60)	.37 (.31)	.28 (.26)
Meta-analytic mean*	0.60	0.27	0.27

SDQ = Strengths and Difficulties Questionnaire. Spearman correlations are given in parenthesis. All correlations significant at the 0.001 level

* From the meta-analysis conducted by Achenbach et al. (1987)

**Table 5:** Inter-rater agreement for SDQ scores.

	Mother and father ratings (n = 3,712)			Mother and teacher ratings (n = 3,574)			Father and teacher ratings (n = 3,100)		
		95% Confidence Interval			95% Confidence Interval			95% Confidence Interval	
SDQ scales	ICC	Lower Bound	Upper bound	ICC	Lower Bound	Upper bound	ICC	Lower Bound	Upper bound
**Emotional symptoms**	.69	.67	.71	.32	.22	.41	.26	.16	.35
**Conduct problems**	.73	.71	.74	.43	.27	.55	.34	.16	.46
**Hyperactivity**	.75	.73	.77	.56	.53	.59	.46	.41	.52
**Peer problems**	.67	.65	.70	.48	.44	.51	.44	.38	.49
**Prosocial behaviour**	.69	.67	.71	.42	.37	.45	.34	.29	.38
**Total difficulties**	.76	.74	.78	.50	.36	.60	.38	.21	.51
Impact*	.68	.67	.71	.42	.38	.46	.37	.32	.41

ICC = Intraclass correlation coefficients. All correlations significant at *P* < 0.001. Results of ICC are calculated using absolute agreement, two-way random-effects model. Interpretations of ICC values: values < 0.40 = poor agreement, values between 0.40 and 0.59 = fair agreement, values between 0.60 and 0.74 = good agreement, values > 0.75 = excellent agreement

* = Assessed with a single question

**Table 6:** Inter-rater agreement for SDQ scores by child age.

	Mother and father ratings			Mother and teacher ratings			Father and teacher ratings		
	ICC (95% Confidence Interval)			ICC (95% Confidence Interval)			ICC (95% Confidence Interval)		
	3-year-olds (n = 1135)	4-year-olds (n = 1103)	5-year-olds (n = 1474)	3-year-olds (n = 1049)	4-year-olds (n = 1105)	5-year-olds (n = 1420)	3-year-olds (n = 920)	4-year-olds (n = 942)	5-year-olds (n = 1238)
**Emotional symptoms**	.72 (.68-.75)	.65 (.60-.69)	.71 (.67-.73)	.27 (.17-.36)	.32 (.20-.42)	.35 (.21-.46)	.19 (.08-.29)	.33 (.20-.43)	.26 (.13-.37)
**Conduct problems**	.69 (.645-.723)	.71 (.67-.74)	.74 (.716-.769)	.37 (.18-.51)	.41 (.23-.53)	.47 (.33-.58)	.26 (.056-.413)	.29 (.132-.414)	.40 (.23-.53)
**Hyperactivity**	.76 (.726-.788)	.73 (.69-.76)	.75 (.725-.78)	.51 (.45-.57)	.57 (.51-.62)	.57 (.52-.61)	.41 (.32-.49)	.50 (.42-.56)	.45 (.37-.52)
**Peer problems**	.65 (.607-.694)	.66 (.611-.694)	.69 (.66-.72)	.48 (.41-.54)	.53 (.47-.59)	.39 (.32-.45)	.48 (.38-.55)	.42 (.34-.50)	.38 (.30-.45)
**Prosocial behaviour**	.68 (.638-.720)	.67 (.626-.706)	.69 (.651-.718)	.35 (.268-.428)	.35 (.265-.421)	.47 (.41-.52)	.27 (.17-.36)	.31 (.22-.40)	.33 (.25-.40)
**Total difficulties**	.76 (.72-.79)	.72 (.69-.75)	.78 (.76-.72)	.43 (.29-.54)	.51 (.35-.62)	.51 (.38-.61)	.33 (.13-.47)	.38 (.22-.51)	.39 (.20-.52)
Impact*	.65 (.61-.69)	.69 (.65-.73)	.70 (.67-.73)	.39 (.31-.46)	.43 (.35-.50)	.43 (.37-.49)	.29 (.19-.39)	.38 (.29-.46)	.40 (.33-.47)

ICC = Intraclass correlation coefficients. All correlations significant at *P*< 0.001. Results of ICC are calculated using absolute agreement, two-way random-effects model. Interpretations of ICC values: Values < 0.40 = poor agreement, values between 0.40 and 0.59 = fair agreement, values between 0.60 and 0.74 = good agreement, values > 0.75 = excellent agreement

* = Assessed with a single question

**Table 7:** Inter-rater agreement for SDQ scores by child gender.

	Mother and father ratings		Mother and teacher ratings		Father and teacher ratings	
	ICC (95% Confidence Interval)		ICC (95% Confidence Interval)		ICC (95% Confidence Interval)	
	Boys (n = 1872)	Girls (n = 1840)	Boys (n = 1835)	Girls (n = 1739)	Boys (n = 1564)	Girls (n = 1536)
**Emotional symptoms**	.70 (.67-.73)	.68 (.65-.71)	.31 (.21-.40)	.34 (.22-.43)	.24 (.13-.34)	.28 (.17-.38)
**Conduct problems**	.74 (.72-.76)	.70 (.67-.73)	.45 (.31-.56)	.39 (.19-.52)	.35 (.20-.47)	.30 (.11-.45)
**Hyperactivity**	.76 (.74-.78)	.73 (.71-.76)	.58 (.54-.62)	.50 (.43-.56)	.52 (.46-.56)	.35 (.25-.43)
**Peer problems**	.71 (.68-.74)	.61 (.57-.65)	.51 (.47-.56)	.40 (.34-.45)	.50 (.43-.56)	.33 (.25-.40)
**Prosocial behaviour**	.68 (.65-.71)	.68 (.64-.71)	.41 (.35-.46)	.36 (.30-.42)	.33 (.26-.40)	.28 (.20-.34)
**Total difficulties**	.78 (.76-.80)	.73 (.70-.76)	.52 (.42-.60)	.44 (.24-.57)	.41 (.26-.53)	.31 (.11-.45)
Impact*	.69 (.66-.72)	.67 (.64-.70)	.46 (.40-.51)	.32 (.25-.39)	.43 (.37-.49)	.22 (.14-.30)

ICC = Intraclass correlation coefficients. All correlations significant at *P*< 0.001. Results of ICC are calculated using absolute agreement, two-way random-effects model. Interpretations of ICC values: Values < 0.40 = poor agreement, values between 0.40 and 0.59 = fair agreement, values between 0.60 and 0.74 = good agreement, values > 0.75 = excellent agreement

* = Assessed with a single question

#### Agreement between mother and father ratings

The highest ICC and Pearson’s correlations were found for the total difficulties scale, the hyperactivity scale and the conduct scale. The lowest ICC and Pearson’s correlations were found for the peer problem scale and the prosocial scale.

The Fisher’s z’ transformation [47] showed that correlations for the hyperactivity, conduct and total difficulties scale were comparable to the meta analytic mean of 0.60 [5], whereas the correlation coefficients for the other subscales were somewhat lower (*p* < 0.001). The total difficulties scale had the highest inter-parent ICC estimate (0.76), indicating excellent agreement. The ICC values for the other subscales and the impact question were good.

#### Agreement between parent and teacher ratings

The highest ICC and Pearson’s correlations were found for the hyperactivity scale and for the peer problems scale, whereas the correlations for the emotional symptom scale were the lowest. Pearson’s correlation coefficients between father and teacher ratings were lower (*p* < 0.001) than the meta-analytic mean of 0.27 for the emotional and the prosocial scale, whereas the correlations were comparable for the other subscales and for the total difficulties score. Pearson’s correlation coefficients between mother and teacher ratings were higher (*p* < 0.001) than the meta-analytic mean of 0.27 in the hyperactivity scale and the total difficulties score, whereas the correlations were comparable for the other subscales.

The Fisher r-to-z transformation showed that for the total scale as well as all the subscales, the ICC estimates between mother and father ratings were significantly higher compared to the ICC values between parent and teacher ratings (*p* < 0.001). ICCs between mother and teacher ratings were significantly higher than those between father and teacher ratings only for the total score and two of the subscales: conduct and hyperactivity (*p* < 0.001). The lowest ICC estimates were found between father and teacher ratings. ICC values for the impact score followed the same pattern, with the highest agreement between mother and father ratings, and the lowest between father and teacher ratings. ICC estimates between mother and teacher ratings were predominantly fair, whereas ICC estimates between father and teacher ratings were predominantly poor.

## Discussion

Methods for identifying children with mental health problems often rely on caregiver’s reports on the child’s functioning. Since children’s behaviour is heavily dependent on the setting [5, 49], assessments of children should be gathered from multiple informants who observe the child in different contexts. However, low inter-rater agreement [5, 11, 30, 50] makes it difficult to perform the clinical assessment based on multiple informants. The aim of the present study was to examine the patterns of inter-rater agreement between parent and teacher SDQ reports of 3–5-year-old children visiting the CHC. Results showed fair or poor agreement between parent and teacher ratings and predominantly good agreement between mother and father ratings. Thus, the findings are consistent with the literature showing low, albeit significant, correlations between parent and teacher reports and higher agreement between mother and father reports.

Low inter-rater agreement is sometimes associated with poor reliability. However, SDQ has shown adequate test-retest reliability and satisfactory internal consistency of the total scales for 4–12-year-olds [11]. Thus, low rates of agreement between informants on SDQ do not necessarily reflect low reliability, but are more likely to be due to children’ situation-specific behaviour [5] and informants’ different standards of judgements. Therefore, the goal when using a structured assessment tool to assess children’s mental health through parent and preschool teacher reports is not to achieve perfect agreement between parent and teacher reports, but rather to get access to their different perspectives.

In the present study, Pearson’s and ICC correlations revealed a pattern of agreement for the subscales, wherein the highest correlations between parents and teachers were found for the hyperactivity and peer problem scale. The highest correlations between mothers and fathers were found for the hyperactivity and conduct scales. This pattern compares favourably with the inter-rater agreement correlations reported in a review by Stone et al. [11] as well as with the inter-parent agreement correlations reported in a study by Davé et al. [26].

Our results indicated different levels of agreement between internalising and externalising behaviours. This finding is consistent with previous research on SDQ [26, 27]. Correlations between parent and teacher ratings were highest for the hyperactivity scale. This finding is also in line with a study reporting inter-rater correlations between parents and teachers in a community sample of children aged 5–6 in the Netherlands [30, 32] and a similar study in Finland [16]. Correlations between parent and teacher ratings were lowest for the emotional problem and prosocial scale, which compares favourably with the inter-rater correlations reported in a review by Stone et al. [11]. A possible explanation for the different levels of agreement is that emotional problems might be more difficult to observe and more influenced by the setting compared to externalising behaviour [51].

The lowest inter-parent correlation was found for the peer problem scale. This finding is in contrast to the findings in a previous study, showing the strongest inter-parent agreement for the peer problem scale [25]. However, in other studies, estimates for the inter-parent agreement for the peer problem scale did not stand out as either the highest or the lowest [11, 26]. The correlation between teacher and parent reports was relatively high for this subscale in our sample. This is not surprising because, given that most Swedish children attend preschool for most part of the day, it is expected that teachers have ample opportunities to observe the child’s peer relationships.

Notable was the finding that the agreement between father and teacher ratings for conduct, hyperactivity and total difficulties was lower (*p* < 0.001) than the agreement between mother and teacher ratings of these scales. The correlations for mother and teacher ratings were closer to the parent—teacher correlations reported in the Stone review [11]. Father and teacher ratings, on the other hand, were significantly lower (*p* < 0.001) than the correlations reported in the review [11] (0.26–0.47), except for the peer problems and the prosocial scale. Furthermore, correlations between mother and teacher ratings in our study were predominantly higher (*p* < 0.001) or equal to the meta-analytic mean of 0.27 [5], while the correlations between father and teacher ratings were predominately lower (*p* < 0.001) or equal to the meta-analytic mean [5]. This is not all that surprising as much of the research conducted on young children uses mothers as informants [52–54]. Thus, most instruments have been developed and standardised for mothers, sometimes leading to problems when using the instrument with fathers [52–54]. The somewhat lower correlations between father and teacher ratings compared to mother and teacher ratings are in accordance with the results in a previous study on inter-rater agreement of behaviour problems in young children, showing higher correlations (r = 0.19) when data were analysed without fathers than with fathers (r = 0.17). This, however, does not mean that fathers are less reliable as informants but probably reflects the lack of fathers as informants in the literature [52–54]. It might also reflect differing parent roles where mothers might have more contact with the preschool teachers or spend more time with the child [55], especially when the child is young.

In the present study, teachers were found to report lower levels of problems compared to both mothers and fathers. In fact, large effect sizes were found between teachers and parents for total difficulties as well as for two of the subscales (emotional symptoms and conduct problems). Findings in a study on teachers’ perspectives on using SDQ in the Swedish preschool setting [56] indicate that the use of structured behavioural assessment tools is highly controversial and that teachers are worried about parents’ reactions and express fear of labelling the child. Consequently, the possibility of teachers underreporting children’s behavioural and emotional problems cannot be excluded in the present study. The finding that teacher’s mean scores were lower than parent’s mean scores is in accordance with a previous study on a sample of normally-developing preschool children in the United States, suggesting that parents report behaviour and emotional problems more frequently than teachers [57]. A study by Brown et al. [58] found that parents of 5–10-year-old children reported a higher proportion of children with conduct problems, but that teachers reported more attention problems than parents. Furthermore, they concluded that gathering teacher ratings increases the number of children needing further evaluation, as agreement on individual children was rather low and single-source information would have led to fewer children with problems being identified.

The present study is part of a comprehensive evaluation of the information sharing, using the SDQ and mainly covers the inter-rater agreement of the method. The first item is the only impact item included in teacher SDQs administered. Hence, for evaluating inter-rater agreement, impact scores could only be generated from this specific question. The ICC estimate for the impact item indicated poor/fair agreement between parent and teacher ratings, while the ICC between mother and father ratings indicated good agreement. In a recent study, SDQs impact supplement (five items) was measured alongside symptoms in children [41]. The results suggest that parent- and teacher-reported impact is a strong predictor of the probability of contact with psychiatric services after 3 years, independent of baseline symptoms [41]. Another reason to measure impact, in addition to symptoms, is that combining the measures might strengthen the complete assessment and lead to valuable discussions between the nurses, parents and teachers.

The sample for the present study was drawn from a trial in which all parents of 3, 4 and 5-year-old children, enrolled at the participating CHCs, were invited to participate. Although the sampling framework catered for a demographically diverse population, the attained sample was not representative of the Swedish population. Participating parents were predominately highly educated, cohabiting and born in Sweden. Thus, our findings cannot be generalised to socio-economically disadvantaged populations. Previous research has indicated that parent-reported behaviour problems correlate with the parent’s level of stress [51, 59] and depression [50, 60, 61] and also with low parental education [30, 62, 63]. Furthermore, associations between socio-economic status, as indicated by education and income, and depression have been established [64]. Differences in education might also have influenced the understanding of the SDQ items. Theoretically, inter-rater agreement in the present study might therefore have been affected by sample characteristics. However, when ICC values were calculated for subgroups of children with parents born outside Sweden, parents not cohabiting and parents without university education, the pattern of correlations were similar to the total sample.

Both parents and teachers who participated in the trial, from which the sample was drawn, had concerns about labelling the child [56]. The questionnaires were used as part of the CHS-routine programme and were therefore not anonymous. This means that the SDQ scores gathered from the parent and teacher reports might have been inadequately low if the informants wanted to avoid stigmatising the child [56], which in turn might have influenced the inter-rater correlations.

The large sample of 3–5-year-olds rated by two or three raters is a strength of the study. A limitation of the study is that Swedish norms for teacher ratings are missing, and although norms for parent ratings are available, they are based on a small sample and not yet published in a peer-reviewed journal. We were therefore unable to evaluate discrepancies in the different informant’s ratings, above cut offs adequate for the context. Also, only one of the items from SDQs impact supplement was administered to teachers. Thus, opportunities to test agreement were limited.

The results from the present study can be used as guidance when deciding whether to obtain reports on a child’s emotional and behavioural functioning from both parents and the child’s preschool teachers. Our results suggest that parents and teachers each provide unique information. However, the results indicate that mother and father reports correlate reasonably and that although inter-parent correlations for the subscales were only good, the total difficulties scale had an ICC estimate of 0.76, indicating excellent agreement.

Conflicting reports have important implications for the nurses’ clinical assessment, and to make sense of the discrepant information nurses must consider the situational demands that different settings place on the child. This is a complex process and providing guidelines presenting calculated intervals of expected parent-teacher (dis)agreement for each subscale might facilitate the nurse’s assessment by making it clearer when and in which subscales the agreement is lower than expected. Nurses should e.g. be aware that higher agreement is expected for the hyperactivity and the peer problem scale, which implies that conflicting reports in these subscales warrant extra attention. The guidelines should also include information about factors that might influence the level of agreement e.g. parent’s gender, parental depression or stress, since such information could be of crucial importance for the nurse’s assessment.

Future research should try to ascertain the reasons as to why the agreement between father and teacher ratings are somewhat lower compared to the agreement between mother and teacher ratings e.g. by matching the reporting parent and teacher by gender.

## Conclusions

The main findings of this study confirmed low, albeit significant, inter-rater agreement between parents’ and teachers’ SDQ ratings. This suggests that correlation alone is not sufficient to judge agreement between different informants. Instead, information from both parents and teachers should be considered when using the SDQ as a method to identify mental health problems in preschool children. However, this also means that clinicians should be comprehensively informed about how to handle the issue of potentially conflicting or incongruent information. The results of this study can be used to provide nurses with guidelines presenting calculated intervals of expected parent-teacher (dis)agreement for each subscale, which may facilitate the nurses’ assessment by making it clearer when and in which subscales the agreement is lower than expected.

Although mothers and fathers each provide unique information about their child’s behaviour, and separate reports should be obtained whenever possible, good inter-parent agreement indicates that a single parent informant may be sufficient if facilitating data collection needs to be prioritised.

## References

[1] Fergusson DM, Horwood LJ, Ridder EM. Show me the child at seven: the consequences of conduct problems in childhood for psychosocial functioning in adulthood. J Child Psychol Psychiatry. 2005;46(8):837–49. 10.1111/j.1469-7610.2004.00387.x 16033632

[2] Hofstra MB, van der Ende J, Verhulst FC. Child and adolescent problems predict DSM-IV disorders in adulthood: a 14-year follow-up of a Dutch epidemiological sample. Journal of the American Academy of Child and Adolescent Psychiatry. 2002;41(2):182–9. 10.1097/00004583-200202000-00012 11837408

[3] Scott S, Knapp M, Henderson J, Maughan B. Financial cost of social exclusion: follow up study of antisocial children into adulthood. BMJ. 2001;323(7306):191 10.1136/bmj.323.7306.191 11473907 PMC35269

[4] Romeo R, Knapp M, Scott S. Economic cost of severe antisocial behaviour in children—and who pays it. The British journal of psychiatry: the journal of mental science. 2006;188:547–53. 16738345 10.1192/bjp.bp.104.007625

[5] Achenbach TM, McConaughy SH, Howell CT. Child/adolescent behavioral and emotional problems: implications of cross-informant correlations for situational specificity. Psychological bulletin. 1987;101(2):213–32. 3562706

[6] The National Board of Health and Welfare. Early interventions to prevent mental health problems among small children (Tidiga insatser mot psykisk ohälsa hos små barn). (In Swedish). www.socialstyrelsen.se; 2013.

[7] Loades M, Mastroyannopoulou K. Teachers’ Recognition of Children’s Mental Health Problems. Child Adolesc Ment Health. 2010;3(15):150–6. 10.1111/j.1475-3588.2009.00551.x 32847228

[8] The Swedish National Agency for Education. Children and staff in preschool autumn 2012 (Barn och personal i förskolan hösten 2012). (In Swedish). www.skolverket.se; 2013 2013-03-14. Contract No.: Dnr. 71–2013:28.

[9] Salari R, Fabian H, Prinz R, Lucas S, Feldman I, Fairchild A, et al The Children and Parents in Focus project: a population-based cluster-randomised controlled trial to prevent behavioural and emotional problems in children. BMC public health. 2013;13:961 10.1186/1471-2458-13-961 24131587 PMC4016486

[10] Goodman R. Psychometric properties of the strengths and difficulties questionnaire. J Am Acad Child Adolesc Psychiatry. 2001;40(11):1337–45. 10.1097/00004583-200111000-00015 11699809

[11] Stone LL, Otten R, Engels RC, Vermulst AA, Janssens JM. Psychometric properties of the parent and teacher versions of the strengths and difficulties questionnaire for 4- to 12-year-olds: a review. Clin Child Fam Psychol Rev. 2010;13(3):254–74. 10.1007/s10567-010-0071-2 20589428 PMC2919684

[12] Malmberg M, Rydell AM, Smedje H. Validity of the Swedish version of the Strengths and Difficulties Questionnaire (SDQ-Swe). Nord J Psychiatry. 2003;57(5):357–63. 10.1080/08039480310002697 14522609

[13] Hunsley J, Mash EJ. Evidence-based assessment. Annu Rev Clin Psychol. 2007;3:29–51. 10.1146/annurev.clinpsy.3.022806.091419 17716047

[14] Mash EJ, Hunsley J. Evidence-based assessment of child and adolescent disorders: issues and challenges. J Clin Child Adolesc Psychol. 2005;34(3):362–79. 10.1207/s15374424jccp3403_1 16026210

[15] Heiervang E, Goodman A, Goodman R. The Nordic advantage in child mental health: separating health differences from reporting style in a cross-cultural comparison of psychopathology. J Child Psychol Psychiatry. 2008;49(6):678–85. 10.1111/j.1469-7610.2008.01882.x 18489678

[16] Borg AM, Kaukonen P, Salmelin R, Joukamaa M, Tamminen T. Reliability of the strengths and difficulties questionnaire among Finnish 4-9-year-old children. Nord J Psychiatry. 2012;66(6):403–13. 10.3109/08039488.2012.660706 22397524

[17] McGraw K, Wong SP. Forming inferences about some Intraclass Correlation Coefficients. Psychological Methods. 1996;1(1):30–46.

[18] De Los Reyes A, Augenstein TM, Wang M, Thomas SA, Drabick DA, Burgers DE, et al The validity of the multi-informant approach to assessing child and adolescent mental health. Psychological bulletin. 2015;141(4):858–900. 10.1037/a0038498 25915035 PMC4486608

[19] Offord DR, Boyle MH, Racine Y, Szatmari P, Fleming JE, Sanford M, et al Integrating assessment data from multiple informants. Journal of the American Academy of Child and Adolescent Psychiatry. 1996;35(8):1078–85. 10.1097/00004583-199608000-00019 8755805

[20] Holmbeck GN, Li ST, Schurman JV, Friedman D, Coakley RM. Collecting and managing multisource and multimethod data in studies of pediatric populations. J Pediatr Psychol. 2002;27(1):5–18. 11726675 10.1093/jpepsy/27.1.5

[21] Strickland J, Hopkins J, Keenan K. Mother-teacher agreement on preschoolers' symptoms of ODD and CD: does context matter? Journal of abnormal child psychology. 2012;40(6):933–43. 10.1007/s10802-012-9622-y 22661105

[22] Goodman R, Meltzer H, Bailey V. The Strengths and Difficulties Questionnaire: a pilot study on the validity of the self-report version. Int Rev Psychiatry. 2003;15(1–2):173–7. 10.1080/0954026021000046137 12745329

[23] Goodman R, Ford T, Corbin T, Meltzer H. Using the Strengths and Difficulties Questionnaire (SDQ) multi-informant algorithm to screen looked-after children for psychiatric disorders. European child & adolescent psychiatry. 2004;13 Suppl 2:II25–31. 15243783 10.1007/s00787-004-2005-3

[24] Goodman R. The Strengths and Difficulties Questionnaire: a research note. J Child Psychol Psychiatry. 1997;38(5):581–6. 9255702 10.1111/j.1469-7610.1997.tb01545.x

[25] Chiorri C, Hall J, Casely-Hayford J, Malmberg LE. Evaluating Measurement Invariance Between Parents Using the Strengths and Difficulties Questionnaire (SDQ). Assessment. 2016;23(1):63–74. 10.1177/1073191114568301 25604631

[26] Dave S, Nazareth I, Senior R, Sherr L. A comparison of father and mother report of child behaviour on the Strengths and Difficulties Questionnaire. Child Psychiatry Hum Dev. 2008;39(4):399–413. 10.1007/s10578-008-0097-6 18266104

[27] Mellor D, Wong J, Xu X. Interparent agreement on the strengths and difficulties questionnaire: a Chinese study. J Clin Child Adolesc Psychol. 2011;40(6):890–6. 10.1080/15374416.2011.614580 22023280

[28] Koo TK, Li MY. A Guideline of Selecting and Reporting Intraclass Correlation Coefficients for Reliability Research. J Chiropr Med. 2016;15(2):155–63. 10.1016/j.jcm.2016.02.012 27330520 PMC4913118

[29] Shrout PE, Fleiss JL. Intraclass correlations: uses in assessing rater reliability. Psychological bulletin. 1979;86(2):420–8. 18839484 10.1037//0033-2909.86.2.420

[30] Mieloo C, Raat H, van Oort F, Bevaart F, Vogel I, Donker M, et al Validity and reliability of the strengths and difficulties questionnaire in 5–6 year olds: differences by gender or by parental education? PloS one. 2012;7(5):e36805 10.1371/journal.pone.0036805 22629332 PMC3356337

[31] Vaz S, Cordier R, Boyes M, Parsons R, Joosten A, Ciccarelli M, et al Is Using the Strengths and Difficulties Questionnaire in a Community Sample the Optimal Way to Assess Mental Health Functioning? PloS one. 2016;11(1):e0144039 10.1371/journal.pone.0144039 26771673 PMC4714886

[32] Mieloo CL, Bevaart F, Donker MC, van Oort FV, Raat H, Jansen W. Validation of the SDQ in a multi-ethnic population of young children. Eur J Public Health. 2014;24(1):26–32. 10.1093/eurpub/ckt100 23867561

[33] Rapee RM, Bogels SM, van der Sluis CM, Craske MG, Ollendick T. Annual research review: conceptualising functional impairment in children and adolescents. J Child Psychol Psychiatry. 2012;53(5):454–68. 10.1111/j.1469-7610.2011.02479.x 22067073

[34] Crone MR, Vogels AG, Hoekstra F, Treffers PD, Reijneveld SA. A comparison of four scoring methods based on the parent-rated Strengths and Difficulties Questionnaire as used in the Dutch preventive child health care system. BMC public health. 2008;8:106 10.1186/1471-2458-8-106 18394152 PMC2359742

[35] Bourdon KH, Goodman R, Rae DS, Simpson G, Koretz DS. The Strengths and Difficulties Questionnaire: U.S. Normative Data and Psychometric Properties. Journal of the American Academy of Child and Adolescent Psychiatry. 2005;44(6):557–64. 10.1097/01.chi.0000159157.57075.c8 15908838

[36] Hawes DJ, Dadds MR. Australian data and psychometric properties of the Strengths and Difficulties Questionnaire. Aust N Z J Psychiatry. 2004;38(8):644–51. 10.1080/j.1440-1614.2004.01427.x 15298588

[37] Woerner W, Becker A, Rothenberger A. Normative data and scale properties of the German parent SDQ. European child & adolescent psychiatry. 2004;13(2):ii3–ii10. 15243780 10.1007/s00787-004-2002-6

[38] Smedje H, Broman JE, Hetta J, von Knorring AL. Psychometric properties of a Swedish version of the "Strengths and Difficulties Questionnaire". European child & adolescent psychiatry. 1999;8(2):63–70. 10435454 10.1007/s007870050086

[39] Ghaderi A, Kadesjö C., Kadesjö B., Enebrink P. The Parental Support Program Joy and Challenges (Föräldrastödsprogrammet Glädje och Utmaningar) (In Swedish). 2014.

[40] Goodman R. The extended version of the Strengths and Difficulties Questionnaire as a guide to child psychiatric caseness and consequent burden. J Child Psychol Psychiatry. 1999;40(5):791–9. 10433412

[41] Stringaris A, Goodman R. The value of measuring impact alongside symptoms in children and adolescents: a longitudinal assessment in a community sample. Journal of abnormal child psychology. 2013;41(7):1109–20. 10.1007/s10802-013-9744-x 23677767 PMC3755220

[42] Goodman R, Iervolino AC, Collishaw S, Pickles A, Maughan B. Seemingly minor changes to a questionnaire can make a big difference to mean scores: a cautionary tale. Soc Psychiatry Psychiatr Epidemiol. 2007;42(4):322–7. 10.1007/s00127-007-0169-0 17334898

[43] Meyers DC, Durlak JA, Wandersman A. The quality implementation framework: a synthesis of critical steps in the implementation process. Am J Community Psychol. 2012;50(3–4):462–80. 10.1007/s10464-012-9522-x 22644083

[44] Dahlberg A, Ghaderi A, Sarkadi A, Salari R. SDQ in the Hands of Fathers and Preschool Teachers-Psychometric Properties in a Non-clinical Sample of 3-5-Year-Olds. Child Psychiatry Hum Dev. 2018. 10.1007/s10578-018-0826-4 PMC6373308 29959588

[45] Cohen J. Statistical Power Analysis for the Behavioral Sciences. New York: NY: Routledge Academic; 1988.

[46] Cicchetti DV. Guidelines, criteria, and rules of thumb for evaluating normed and standardized assessment instruments in psychology. Psychol Assess. 1994;6(4):284–90.

[47] Fisher RA. On the probable error of a coefficient of correlation deduced from a small sample. Metron. 1921(1):3–32.

[48] IBM C. IBM SPSS Statistics for Windows. 22 ed. Armonk, New York2013.

[49] Goodman R, Renfrew D, Mullick M. Predicting type of psychiatric disorder from Strengths and Difficulties Questionnaire (SDQ) scores in child mental health clinics in London and Dhaka. European child & adolescent psychiatry. 2000;9(2):129–34. 10926063 10.1007/s007870050008

[50] Gross D, Sambrook A., Fogg L. Behavior problems among young children in low-income urban day care centers. Res Nurs Health. 1999(22):15–25. 9928960 10.1002/(sici)1098-240x(199902)22:1<15::aid-nur3>3.0.co;2-2

[51] Kolko DJ, Kazdin AE. Emotional/behavioral problems in clinic and nonclinic children: correspondence among child, parent and teacher reports. J Child Psychol Psychiatry. 1993;34(6):991–1006. 8408380 10.1111/j.1469-7610.1993.tb01103.x

[52] Phares V. Where's poppa? The relative lack of attention to the role of fathers in child and adolescent psychopathology. Am Psychol. 1992;47(5):656–64. 1642375 10.1037//0003-066x.47.5.656

[53] Costigan CL, Cox MJ. Fathers' participation in family research: is there a self-selection bias? J Fam Psychol. 2001;15(4):706–20. 11770476 10.1037//0893-3200.15.4.706

[54] Woollett A, White D., Lyon M. Studies involving fathers: Subject refusal, attrition, and sampling bias. Curr Psychol Reviews. 1982(2):193–212.

[55] Statistics Sweden. Women and men in Sweden 2014 (Pa° tal om kvinnor och män) (In Swedish) Örebro: SCB-Tryck; 2014.

[56] Falt E, Sarkadi A, Fabian H. Exploring Nurses', Preschool Teachers' and Parents' Perspectives on Information Sharing Using SDQ in a Swedish Setting—A Qualitative Study Using Grounded Theory. PloS one. 2017;12(1):e0168388 10.1371/journal.pone.0168388 28076401 PMC5226714

[57] Winsler A, Wallace GL. Behavior Problems and Social Skills in Preschool Children: Parent-Teacher Agreement and Relations with Classroom Observations. Early Educ Dev. 2002(13):41–58.

[58] Brown J, Wissow LS., Gadomski A.,Zachary C., Bartlett E., Horn I Parent and Teacher Mental Health Ratings of Children Using Primary-Care Services: Interrater Agreement and Implications for Mental Health Screening. Ambul Pediatr. 2006(6):347–51. 17116609 10.1016/j.ambp.2006.09.004 PMC1761112

[59] De Los Reyes A, Kazdin AE. Measuring informant discrepancies in clinical child research. Psychol Assess. 2004;16(3):330–4. 10.1037/1040-3590.16.3.330 15456389

[60] Richter JE. Depressed mothers as informants about their children: a critical review of the evidence for distortion. Psychological bulletin. 1992(112):485–99. 1438639 10.1037/0033-2909.112.3.485

[61] De Los Reyes A, Kazdin AE. Informant discrepancies in the assessment of childhood psychopathology: a critical review, theoretical framework, and recommendations for further study. Psychological bulletin. 2005;131(4):483–509. 10.1037/0033-2909.131.4.483 16060799

[62] Graves S, Blake J., Kim ES. Differences in Parent and Teacher Ratings of Preschool Problem Behavior in a National Sample: The Significance of Gender and SES. J Early Interv. 2012(34):151–65.

[63] Fuchs S, Klein AM., Otto Y. Prevalence of Emotional and Behavioral Symptoms and their Impact on Daily Life Activities in a Community Sample of 3 to 5-Year-Old Children. Child Psychiatry Hum Dev. 2012(44):493–503. 10.1007/s10578-012-0343-9 23111504

[64] Lorant V, Deliege D, Eaton W, Robert A, Philippot P, Ansseau M. Socioeconomic inequalities in depression: a meta-analysis. Am J Epidemiol. 2003;157(2):98–112. 12522017 10.1093/aje/kwf182

